# Use of Benzodiazepines in Chemical Submission in Children: Insights From Electroencephalography and Toxicological Analysis

**DOI:** 10.7759/cureus.76940

**Published:** 2025-01-05

**Authors:** Sahar Amrani Hanchi, Sanae Achour, Hasnae Hoummani, Sana Chaouki

**Affiliations:** 1 Pediatrics Department, Hassan II University Hospital, Faculty of Medicine and Pharmacy, Fez, MAR; 2 Biomedical and Translational Research Laboratory, Hassan II University Hospital, Faculty of Medicine and Pharmacy, Fez, MAR; 3 Toxicology Department, Hassan II University Hospital, Faculty of Medicine and Pharmacy, Fez, MAR; 4 Pediatric Neurology Department, Hassan II University Hospital, Faculty of Medicine and Pharmacy, Fez, MAR

**Keywords:** benzodizepines, chemical submission, electroencephalography (eeg), high performance liquid chromatography ( hplc), seizures, toxicological analysis

## Abstract

This case report identifies benzodiazepine intoxication as the cause of seizures in a pediatric patient, a diagnosis that is often missed in similar clinical presentations. The recent deaths of two siblings under similar circumstances make this case particularly unique, emphasizing the need to consider toxicological causes in such contexts. Additionally, the report highlights the crucial role of EEG and advanced toxicological analysis, like high-performance liquid chromatography (HPLC), in diagnosing and managing complex cases, underscoring their importance in improving patient outcomes. We describe the case of a 12-year-old child who was admitted to the pediatric emergency department of Hassan II University Hospital in Morocco for a brief apyretic convulsive seizure with loss of consciousness. The biological analysis, brain MRI, and lumbar puncture (LP) analysis were normal. Notably, the patient had a concerning family history with the recent deaths of two younger siblings, aged five and six years, occurring 20 days apart. These deaths were preceded by similar neurological symptoms. Over several weeks, the patient continued to experience epileptic seizures. Electroencephalography (EEG) findings suggested benzodiazepine intoxication. Subsequent toxicological investigations revealed significantly elevated benzodiazepine levels in both serum and urine, confirmed by HPLC. This case underscores the critical need for considering EEG patterns and toxicological analysis in pediatric patients with unexplained seizures, particularly in the context of a suspicious family history that suggests potential child maltreatment. Early identification and confirmation of benzodiazepine intoxication can be life-saving and may prevent further tragic outcomes.

## Introduction

Chemically induced submissions are a major pediatric public health issue. Apart from medical prescriptions, the administration of psychoactive substances to children is a specific form of chemical submission, usually aimed at obtaining sedation [1]. Children's chemical abuse is difficult to diagnose because, in most cases, the events occur in the family environment [2]. Moreover, a "good" compound that can be used to commit a drug-facilitated crime usually possesses a short elimination half-life and amnesic properties so that the victim is less able to accurately recall the circumstances under which the offense occurred [3]. A large variety of medications are used for chemical submission [4-6]. Mainly benzodiazepines due to their rapid onset of action, disinhibition, muscle relaxation, and anterograde amnesia that they produce. Chemical submission often reveals the sudden appearance of neuropsychiatric disorders in children. We present a case of a drug-facilitated crime due to maltreatment of children by their mother presenting psychiatric and behavioral disorders in order to obtain sedation, and in which bromazepam poisoning was revealed by electroencephalography (EEG), determined and confirmed by specific and sophisticated high-performance liquid chromatography procedures.

## Case presentation

Our patient is a healthy 12-year-old Moroccan female, born to non-consanguineous parents, admitted with a two-month history of epileptic seizures. Her development was normal. Her family history was unremarkable until four months ago.

Two siblings, aged five and six years, died two and three months ago, respectively. The sibling had a history of recent generalized tonic-clonic seizures. An EEG, computed tomography, and biological analysis proved to be normal. They were subsequently treated with valproic acid and phenobarbital. They were admitted to an intensive care unit for status epilepticus. The treatment was unsuccessful, and death occurred a few hours later.

Our patient had a total of four seizures. According to the patient's history, she was initially admitted to a regional hospital where physical examination was reported as normal. Biological analysis and CT of the head were performed with all normal results. However, toxic screening was not conducted. She was then referred to the Pediatric Neurology Department. Behavioral, mental, and consciousness disorders, vomiting, apyretic convulsive seizures, and muscular weakness were noted on physical examination.

The biological analysis, brain MRI, EEG, and lumbar puncture (LP) were performed. No electrolyte imbalances were found. Renal function test, test for current infection (cytobacteriological urine examination and C-reactive protein), LP, brain MRI, and chest X-ray were all normal (see Table 1). 

**Table 1:** Comprehensive biological and radiological analysis CPK-MB - creatine phosphokinase-MB; ECBU - extended centrifugal blood unit

Category	Parameter	Result	Unit	Normal range
Hematological parameters	Hemoglobin (Hb)	13.5	g/dL	12-16 (females), 13-17 (males)
	White blood cells (WBC)	10,050	/mm³	4,000-11,000
	Platelets (Plt)	352,000	/mm³	150,000-450,000
	Prothrombin time (PT)	100%	%	70-100
Electrolytes and biochemical parameters	Calcium (Ca)	80	mg/L	80-100
	Potassium (K⁺)	3.9	mmol/L	3.5-5.0
	Sodium (Na⁺)	133	mmol/L	135-145
	Urea	0.34	g/L	0.15-0.45
	Glucose (glycemia)	1	g/L	0.7-1.1
	Creatinine	4	mg/L	6-12 (adults, females)
Liver and muscle enzymes	Aspartate aminotransferase (ASAT)	46	U/L	10-40
	Alanine aminotransferase (ALAT)	25	U/L	7-56
	Creatine phosphokinase (CPK)	81	U/L	26-192
	CPK-MB	30	U/L	<25 (may vary slightly by lab)
Inflammatory markers	C-reactive protein (CRP)	1	mg/L	<10
Microbiological and functional tests	Lumbar puncture (LP)	Negative	-	No signs of infection
	Urine culture (ECBU)	Negative	-	No bacterial growth
	Liver function tests	Normal	-	Normal enzyme levels
Radiological findings	Brain MRI	Normal	N/A	No abnormalities detected
	Chest X-ray	Normal	N/A	No abnormalities detected

**Figure 1:**
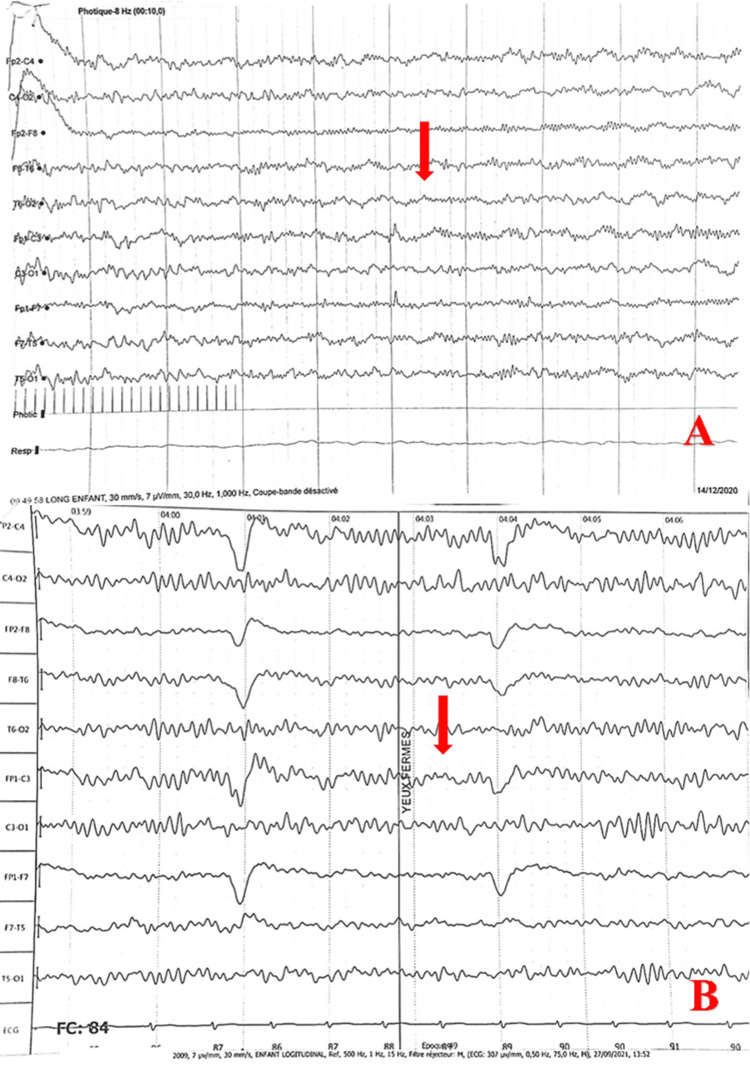
Electroencephalographic beta activity during bromazepam exposure and resolution post-event A: Electroencephalography (EEG) in the patient exposed to bromazepam revealed excessive beta activity, characterized by a recording speed of 30 mm/s, sensitivity of 7 µV/mm, frequency of 30.0 Hz, and filter settings at 1.000 Hz. B: A follow-up EEG performed post-event demonstrated a return to normal activity with no observed abnormalities under identical parameters (30 mm/s, 7 µV/mm, 30.0 Hz, 1.000 Hz).

Infectious, renal, cardiac, hepatic, and central nervous system origins were ruled out according to the laboratory results mentioned above. Genetic etiology was less evident, as the epileptic signs occurred at different ages in both sisters. Interestingly, a subsequent EEG showed high-frequency basal activity and increased Beta activity in all states of vigilance that can evoke a pharmacological origin (benzodiazepines), although the patient was under phenobarbital and valproate sodium, pointing towards the toxic origin of the disease (Figure 1). 

**Figure 2:**
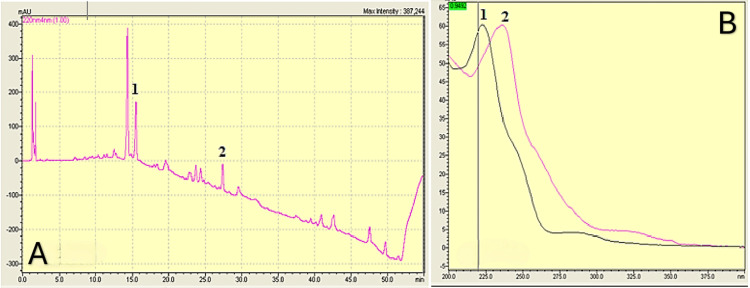
Chromatographic identification of bromazepam: sample peak and database comparison A: Chromatogram illustrating the bromazepam peak: 1 - bromazepam with a retention time (RT) of 15.50 minutes; 2 - the internal standard "methyl-clonazepam" with an RT of 27.39 minutes. B: Comparison of the bromazepam peak: 1 - extracted bromazepam peak from the sample; 2 - corresponding bromazepam peak from the spectral database (library).

After several investigations with the parents, they denied having given any medicine to the child. Nevertheless, toxicological analyses of urine and blood were conducted on day 1 to detect and confirm the presence of benzodiazepine in both samples. All toxicological investigations of blood and urine samples were positive for benzodiazepines - the rapid test, enzyme-multiplied immunoassay technique (EMIT) assay, with a concentration of 71.7µg/L, and high-performance liquid chromatography with diode-array detection (HPLC-DAD) analyses revealed a high plasma peak of bromazepam, triggering the alert (Figure 2). 

Our patient's clinical condition improved after symptomatic treatment, and laboratory tests normalized within about 24 hours. Control immunoenzymatic tests of blood and urine on days 2 and 4 of hospitalization showed the presence of benzodiazepines at levels of 46.6µg/L and 26.2 µg/L, respectively, which are below the cut-off value of 50 µg/L. A judicial alert was issued to highlight the possibility of a criminal act. Further investigations revealed that the mother, who had a history of behavioral and mental disorders, was the perpetrator in those cases. It was discovered that she had administered her prescribed sedative medication, bromazepam, to the child. The mother showed little interest in caring for her children and did not dispute her actions during the investigation.

## Discussion

Chemical submission is not a recent phenomenon. Besides the classic robbery cases and sexual assault, there has been a notable increase in incidents where drugs are administered solely to induce sedation, particularly in pediatric populations, causing public alarm [1]. In Morocco, this phenomenon remains largely hypothetical due to a lack of available data. Benzodiazepines and hypnotics are the most frequently used for drug-facilitated crimes, as reported in many studies [7-9]. Additionally, methadone was detected in babies who were administered the drug for relaxation [5].

Benzodiazepine toxicity in children can be fatal. Usually, it induces mild to moderate central nervous system depression. Deep coma requiring assisted ventilation is rare in pediatric cases [8]. Most children exhibit serious symptoms within four hours of ingestion. Ataxia is the most common sign of toxicity, occurring in 90% of pediatric patients, while respiratory compromise occurs in less than 10% of cases [4], and seizures can also occur [10,11].

Despite the clinical signs observed in our patient were fully in line with a benzodiazepine intoxication, the situation was complex and unusual. The clinical symptoms were ambiguous, and both sisters had previously died after experiencing several epileptic seizures at different ages, even though all biological parameters were normal. Management of this case included proceeding with elimination tests. The brain MRI examination was normal, ruling out cerebral organic or anatomic pathology. The genetic etiology was deemed less likely, as genetic diseases typically manifest at the same age in siblings. In this case, the deceased sisters presented similar symptoms but were triggered at different ages. Furthermore, all biological analyses ruled out any infectious, renal, hepatic, or cardiac etiology of the disease. This pointed to a toxic etiology.

The EEG recording, indeed, prompted an investigation for toxic substances, specifically benzodiazepines. Numerous drugs have been shown to alter EEG patterns, and many of these drug-induced changes are clinically significant. In our patient, the EEG showed increased beta activity, indicative of benzodiazepine poisoning. Literature supports that benzodiazepines, similar to barbiturates, significantly increase beta activity [11,12]. High blood levels can also result in the slowing of background activity. Figure 2 from our patient illustrates an accelerated pattern, suggesting benzodiazepine intoxication due to altered beta activity without other abnormalities.

These findings prompted toxicological investigations, including urine and blood analyses, which revealed the presence of benzodiazepines at a concentration of 71.7 µg/L. The specific benzodiazepine was identified as bromazepam through HPLC-DAD screening, which showed a high plasma peak. Bromazepam, a benzodiazepine commonly used as an anti-anxiety agent, is typically administered in single oral doses ranging from 3 to 12 mg [13]. Therapeutic serum concentrations of bromazepam are in the range of 80-170 µg/L [13], with levels higher than 250 µg/L considered potentially toxic. The delay in management may explain the non-toxic dose obtained in our patient, suggesting a likely repeated drug intake over a long period. Sudden withdrawal of bromazepam can lead to an increase in epileptiform activity and even seizures [14-21]. The signs presented in the reported observation are consistent with moderate acute intoxication by bromazepam. Our patient experienced repeated convulsive seizure episodes, unconsciousness, drowsiness, and confusion, which align with data from the literature [14].

Psychologically, long-term use of benzodiazepines may lead to loss of self-confidence, social phobia, and varying degrees of mental disorders and retardation. A study on the effect of benzodiazepines in children whose seizures were resistant to most other antiepileptic drugs showed that mental retardation was present in 80% of the children, and 62.5% had Lennox-Gastaut syndrome [10]. Dysmorphism and mental retardation have also been reported in seven Swedish children born to mothers who had taken high doses of benzodiazepines regularly during pregnancy [17]. This may explain the abnormal behaviors and the mental retardation presented by the patient. The classic presentation of patients with isolated benzodiazepine overdose includes central nervous system depression with normal or near-normal vital signs. Classic symptoms include slurred speech, ataxia, and altered mental status [18,20].

Although flumazenil is the only antidote for benzodiazepine poisoning, it was not used in our case. Treatment was limited to seizure control with valproate sodium/phenobarbital, respiratory resuscitation, and vascular filling. The evolution of bromazepam intoxication, with well-conducted treatment, is generally favorable within 24-48 hours, usually without sequelae [6-8]. However, certain complications can arise, such as poor concentration, drowsiness, dysarthria, ataxia, motor incoordination, muscle weakness, diplopia, vertigo, and mental confusion, especially in cases of repeated intake [15,16]. Deaths caused by bromazepam alone, in the absence of other xenobiotics or pathology, are uncommon, although some fatal cases have been reported in the literature [21,22]. The risk of developing significant withdrawal symptoms is related to dosage and duration of exposure. In our case, the determined dose (71.7 µg/L) was not fatal, and the outcome was relatively benign. The patient's condition improved within 48 hours of admission; the child was stable, conscious, apyretic, seizure-free, and fully alert, with no other abnormalities. A psychiatric and psychological follow-up was carried out for both the patient and her mother.

## Conclusions

Chemical submission often presents with the sudden onset of neuropsychiatric disorders in children, typically manifesting as an atypical clinical presentation. In such cases, toxic ingestion or exposure should immediately be considered in the differential diagnosis. Specific clues in the clinical presentation or patient history, such as unexplained neurological symptoms or recent exposure to potential toxins, may allow for early suspicion and consideration of toxic exposure. Early detection is critical as it enables efficient treatment and prevents unnecessary harm caused by delayed identification of the seizure etiology.

The importance of early suspicion is further highlighted in this case, particularly given the recent deaths of two siblings, which underscores the need for heightened vigilance in similar scenarios. This case report underscores the crucial importance of utilizing EEG to evoke the possibility of a toxic etiology. Furthermore, drug screening using HPLC methods has become increasingly significant, not only for the rapid detection of toxic substances but also for uncovering potential medico-legal implications. Early recognition and diagnostic precision are vital in improving outcomes, guiding timely intervention, and addressing the broader medico-legal aspects of such cases.
